# Associations of reproductive breast cancer risk factors with breast tissue composition

**DOI:** 10.1186/s13058-021-01447-2

**Published:** 2021-07-05

**Authors:** Lusine Yaghjyan, Rebecca J. Austin-Datta, Hannah Oh, Yujing J. Heng, Adithya D. Vellal, Korsuk Sirinukunwattana, Gabrielle M. Baker, Laura C. Collins, Divya Murthy, Bernard Rosner, Rulla M. Tamimi

**Affiliations:** 1 College of Public Health and Health Professions and College of Medicine, Department of Epidemiology, University of Florida, 2004 Mowry Rd, Gainesville, FL 32610 USA; 2 Division of Health Policy and Management, College of Health Sciences, Korea University, Seoul, South Korea; 3 Department of Public Health Sciences, Graduate School, Korea University, Seoul, South Korea; 4 Department of Pathology, Harvard Medical School, Beth Israel Deaconess Medical Center, Boston, MA USA; 5 Institute of Biomedical Engineering (IBME), Department of Engineering Science, Old Road Campus Research Building, University of Oxford, Oxford, UK; 6 Channing Division of Network Medicine, Department of Medicine, Brigham and Women’s Hospital and Harvard Medical School, Boston, MA USA; 7 Department of Population Health Sciences, Weill Cornell Medicine, New York, NY USA

**Keywords:** Benign breast disease, Parity, Breastfeeding, Age at first child, Breast cancer risk

## Abstract

**Background:**

We investigated the associations of reproductive factors with the percentage of epithelium, stroma, and fat tissue in benign breast biopsy samples.

**Methods:**

This study included 983 cancer-free women with biopsy-confirmed benign breast disease (BBD) within the Nurses’ Health Study and Nurses’ Health Study II cohorts. The percentage of each tissue type (epithelium, stroma, and fat) was measured on whole-section images with a deep-learning technique. All tissue measures were log-transformed in all the analyses to improve normality. The data on reproductive variables and other breast cancer risk factors were obtained from biennial questionnaires. Generalized linear regression was used to examine the associations of reproductive factors with the percentage of tissue types, while adjusting for known breast cancer risk factors.

**Results:**

As compared to parous women, nulliparous women had a smaller percentage of epithelium (β = − 0.26, 95% confidence interval [CI] − 0.41, − 0.11) and fat (β = − 0.34, 95% CI − 0.54, − 0.13) and a greater percentage of stroma (β = 0.04, 95% CI 0.01, 0.08). Among parous women, the number of children was inversely associated with the percentage of stroma (β per child = − 0.01, 95% CI − 0.02, − 0.00). The duration of breastfeeding of ≥ 24 months was associated with a reduced proportion of fat (β = − 0.30, 95% CI − 0.54, − 0.06; p-trend = 0.04). In a separate analysis restricted to premenopausal women, older age at first birth was associated with a greater proportion of epithelium and a smaller proportion of stroma.

**Conclusions:**

Our findings suggest that being nulliparous as well as having a fewer number of children (both positively associated with breast cancer risk) is associated with a smaller proportion of epithelium and a greater proportion of stroma, potentially suggesting the importance of epithelial-stromal interactions. Future studies are warranted to confirm our findings and to elucidate the underlying biological mechanisms.

**Supplementary Information:**

The online version contains supplementary material available at 10.1186/s13058-021-01447-2.

## Background

Breast cancer remains the most commonly diagnosed cancer in women in the USA and worldwide [1]. The vast majority of breast tumors are carcinomas that arise from the breast epithelium. Sarcomas of the breast are exceedingly rare and are thought to originate from stromal components of the breast (< 1% of all breast tumors) [2]. It has long been recognized that women with a greater proportion of fibroglandular breast tissue (combined epithelium and stroma) as reflected on a mammogram (also referred to as breast density) are at a greater risk of breast cancer [3]. While several breast cancer risk factors are suggested to influence breast tissue composition and thus subsequent breast cancer risk, the epidemiological evidence on these relationships remains very limited.

Reproductive factors related to childbearing are also recognized as breast cancer risk factors. Parity, younger age at first birth, and breastfeeding are associated with reduced breast cancer risk [4–8]. A longer period between menarche and first pregnancy, on the other hand, is associated with increased breast cancer risk [9–12]. Whether any of these factors could influence adult breast tissue composition is unclear.

Some previous studies of associations between reproductive factors and mammographic breast density, a well-established strong risk factor reflective of relative amounts of fibroglandular vs. fatty tissue content on the mammogram, found inverse associations of parity and positive associations of age at first birth and duration of breastfeeding with breast density [13–18]. In our recent study of reproductive factors and breast density, parous women as compared to nulliparous women had lower percent breast density (proportion of fibroglandular tissue out of the total breast area), smaller absolute dense area (area of fibroglandular tissue), and greater non-dense area (area of adipose tissue). The positive associations of breastfeeding with absolute dense and non-dense areas were limited to premenopausal women, while the positive association of the age at first child’s birth with percent density and the inverse association with the non-dense area were limited to postmenopausal women [19]. Despite this evidence on the associations of reproductive factors with tissue composition on mammograms, only a few studies have examined these associations using direct measurement of tissue components in the non-malignant breast tissue of cancer-free women. Gabrielson et al. found positive associations of parity and duration of breastfeeding with epithelial area [20], while an earlier study on associations of reproductive factors with the proportion of epithelium or stroma found no such associations [21].

In this study, we aimed to assess the associations of several reproductive variables (parity, age at first birth, breastfeeding, time since last pregnancy, and duration of the time between menarche and first birth) with the extent of epithelial, stromal, fibroglandular, and fat tissue in non-malignant breast tissue from benign breast biopsy samples using prospective data in cancer-free women from the Nurses’ Health Study (NHS) and Nurses’ Health Study II (NHSII) and a deep-learning computational pathology method for tissue composition assessment.

## Materials and methods

### Study population

Our analysis included cancer-free women (controls) from the nested case-control study of breast cancer conducted among the subcohort of women with biopsy-confirmed benign breast disease (BBD) in the NHS and NHSII cohorts [22, 23]. These prospective cohorts followed registered nurses in the USA who were 30–55 years (NHS) or 25–42 years old (NHSII) at enrollment. After the administration of the initial questionnaire, the information on breast cancer risk factors (body mass index [BMI], reproductive history, postmenopausal hormone [PMH] use, and alcohol use) and any diagnoses of cancer or other diseases (including BBD) was updated through biennial questionnaires which were then confirmed via medical record review [13, 24]. Details of this nested case-control study and the BBD assessment have been previously described [22, 23].

Early NHS questionnaires (1976, 1978, and 1980) asked whether the participant had ever been diagnosed with “fibrocystic disease” or “other BBD” and whether she had been hospitalized in relation to this diagnosis. Beginning in 1982, the NHS questionnaires specifically asked about a history of biopsy-confirmed BBD (fibrocystic disease or other BBD). The initial 1989 NHS II questionnaire and all subsequent biennial questionnaires also asked the participants to report any diagnosis of BBD and to indicate whether it was confirmed by biopsy or aspiration.

Cases were women with biopsy-confirmed BBD who reported a diagnosis of breast cancer during 1976–1998 for the NHS and 1989–1999 for the NHSII following their BBD diagnosis. Using incidence density sampling, four women with biopsy-confirmed BBD who were free of breast cancer at the time of the matching case’s diagnosis (controls) were matched to the respective case on the year of birth and year of benign breast biopsy [25]. We attempted to obtain BBD pathology records and archived biopsy specimens for all cases and controls from their hospital pathology departments; our ability to obtain biopsy blocks did not significantly differ by case and control status. Women were excluded if they had evidence of in situ or invasive carcinoma or unknown lesion type at the time of benign breast biopsy (22 cases and 12 controls). Only controls from this nested case-control study were used to examine the associations of reproductive factors with the extent of different tissue types. Out of 1907 controls, 983 had tissue readings, and information on reproductive factors was included in this analysis. Women with and without available tissue readings (as explained under the “Whole-slide image acquisition” section) had similar distributions of breast cancer risk factors.

The study protocol was approved by the institutional review boards of the Brigham and Women’s Hospital and Harvard T.H. Chan School of Public Health and those of participating registries as required. Consent was obtained or implied by the return of questionnaires.

### Benign breast biopsy confirmation and BBD subtypes

Hematoxylin and eosin (H&E) breast tissue slides were retrieved for biopsy-confirmed BBD patients who gave permission to review their biopsy records. The slides were independently reviewed by one of three pathologists in a blinded fashion, i.e., the evaluating pathologists were blinded to the type of BBD noted on the original diagnosis [26, 27]. Any slide identified as having either questionable atypia or atypia was jointly reviewed by two pathologists. For each set of slides, a detailed worksheet was completed, and the benign breast biopsy was classified according to the categories of Page et al. [28] as non-proliferative, proliferative without atypia, or atypical hyperplasia (ductal or lobular hyperplasia) [22].

### Whole-slide image acquisition

H&E slides were digitized into whole-slide images at × 20 (n = 93) or × 40 (n = 890) using the Panoramic SCAN 150 (3DHISTECH Ltd., Budapest, Hungary). For women with good-quality slides, up to six slides from different tissue blocks were digitized. H&E slides that were not digitized were due to poor quality, slides too thick to fit into the scanner, and plastic mounting coverslips. Attempts to create new H&E slides were not always possible due to missing (or returned to hospital) blocks, old style blocks not created using tissue cassettes, or poor-quality blocks [29]. Slides were successfully digitized for approximately 80% of all control women in the original nested case-control study.

### Quantification of epithelium, stroma, and fat

Whole-slide images were processed using a deep-learning computational pathology method to segment BBD tissues into epithelial, stroma, and fat regions. Tissue image analysis included normal terminal duct lobular units (TDLUs) and BBD lesions (referred to as “non-malignant” throughout this manuscript). Details of the image analysis method and its performance are described elsewhere [30]. Briefly, to evaluate the tissue segmentation network, precision, recall, and Dice similarity coefficient were calculated using the held-out test set (n = 48). Dice similarity coefficient is the harmonic mean of precision (i.e., sensitivity) and recall (i.e., positive predictive value) and assesses how accurate the automated segmentation compares with ground truth on a pixel-wise basis. The range for Dice similarity coefficient is from 0 to 1, with 1 indicating perfect overlap. The majority of the precision, recall, and Dice similarity coefficient values of the tissue segmentation network and nuclei detection were > 0.75 [30]. For more details about the nuclear segmentation network, please refer to the previously published methods paper by Vellal et al. [30].

For each whole-slide image, our method computed total, epithelial, stromal, and adipose tissue areas in pixels. We next calculated the average percent of each tissue type out of the total area across all available slides for each woman (median = 3, range 1–4), weighted by the total tissue area of the slides. We examined the associations of reproductive factors with the percentage of each of these individual tissue regions as well as combined epithelial and stromal tissue (fibroglandular area).

### Reproductive variables

The data on age at menarche, parity, age at first birth, and breastfeeding were available from baseline and biennial questionnaires, completed closest to the date of the biopsy. Among all eligible controls with tissue readings, the completeness of the data on parity was 98.3%. Among parous women with tissue readings, information on age at first birth and breastfeeding was available for 98.4% and 94.1% of the sample, respectively. Age at menarche was available for 99.6% of the sample. For 4 women with missing age at menarche, a median value in the study sample was imputed, as done in previous studies [31–33]. Time since the last pregnancy was available for 86.3% of the study sample.

Age at first birth was categorized as < 25, 25–29, and ≥ 30 years. Parity was defined both as a binary variable (nulliparous, parous) as well as categorical (1, 2, 3, and ≥ 4 children). Additionally, the number of children among parous women was modeled as a continuous variable. Age at first birth was modeled both as a categorical (< 25, 25–29, and ≥ 30 years) and as a continuous variable. Information on breastfeeding was collected one time (on the 1986 questionnaire), and women were asked to report the total months of breastfeeding. Lifetime duration of breastfeeding (sum of breastfeeding duration across all births) was classified as none to < 1, 1 to < 12, 12 to < 24, and ≥ 24 months. Age at menarche was modeled both as a categorical (< 12, 12, 13, and > 13 years) and as a continuous variable. The time interval between menarche and first birth as well as the time since the last pregnancy was modeled as continuous variables.

### Covariate information

Information on breast cancer risk factors was obtained from the biennial questionnaires closest to the date of the biopsy. Women were considered to be postmenopausal if they reported (1) no menstrual periods within the 12 months before biopsy with natural menopause, (2) bilateral oophorectomy, or (3) hysterectomy with one or both ovaries retained and were 54 years or older for ever smokers or 56 years or older for never smokers [34, 35].

### Statistical analysis

We used multivariate linear regression to examine the associations of parity, age at first birth, breastfeeding, and the interval between menarche and first birth with the proportion of epithelial, stromal, fibroglandular, and fat tissues. Because tissue type measures were non-normally distributed, we used log-transformed values in all the regression analyses to improve normality. The risk estimates were adjusted for age (continuous), body mass index (BMI, continuous), a family history of breast cancer (yes vs. no), alcohol use (none, > 0 to < 5, ≥ 5 g/day), age at menarche (< 12, 12, 13, > 13), menopausal status/postmenopausal hormone use (pre-, post-/no hormones, post-/past hormone use, post-/current hormone use, post-/unknown hormone use status), and study cohort (NHS, NHSII). Additionally, in the analysis of the association of breastfeeding, the estimates were adjusted for parity and age at first birth. In the analysis of the associations of parity and age at first birth, the risk estimates were mutually adjusted for these two variables. In the analysis for the interval between menarche and first birth, the estimates were adjusted for parity.

The analyses of all reproductive variables except nulliparity and age at menarche were limited to parous women only. Parity, age at first birth, and age at menarche were modeled both as continuous and categorical, and breastfeeding was modeled as categorical. The lowest category for parity (1 child), age at first birth (< 25 years), and breastfeeding (0 to < 1 month) were used as the reference. To assess the overall trend for each of the categorical reproductive variables, we used respective medians within each category. The time since the last pregnancy as well as the duration of the interval between menarche and first birth were modeled as continuous variables. As in our prior study on reproductive factors and mammographic breast density, some of the associations were limited to either pre- or postmenopausal women; in the secondary analysis, we examined these associations separately in premenopausal women; the small sample of postmenopausal women (n = 290) in our study did not allow us to draw meaningful conclusions for this stratum. Finally, to account for the potential influence of BBD lesions on the study findings, we additionally adjusted all models for the type of the BBD.

In addition to the main approach, we used the SAS Proc Glimmix procedure which accounts for non-normal data distributions to examine the associations with tissue types using their original continuous scale. The analyses were performed using the SAS software (version 9.4, SAS Institute, Cary, NC, USA).

## Results

In this study of 983 cancer-free women, 299 (30.4%) had non-proliferative disease, 559 (56.9%) had proliferative disease without atypia, and 125 (12.7%) had atypical hyperplasia, consistent with previously reported distributions of these BBD subtypes [27]. The distribution of different tissue types across these subtypes in our study is presented in Supplementary Table 1. The average proportion of epithelium, stroma, and fat in our study sample was 9.1% (range 0.5–52.2%), 72.4% (range 23.6–99.0%), and 18.5% (range 0–71.3%), respectively.

In our study sample, the average age at the biopsy was 42 years (range 19–58 years). A majority of the women were premenopausal at the biopsy (62.3%). The majority of women were parous (89.9% for premenopausal and 92.8% for postmenopausal), and the majority of parous women had at least two children (87.0% for premenopausal and 93.7% for postmenopausal) and breastfed for at least 1 month (58.8% for premenopausal and 50.4% for postmenopausal). The average age at first birth was 25 years (range 15–40 years) for premenopausal women and 25 years (range 16–37 years) for postmenopausal women. Age-adjusted characteristics of pre- and postmenopausal women in the study by nulliparous status are presented in Table 1.

**Table 1:** Age-adjusted characteristics of women at the time of the biopsy, stratified by menopausal status and parity

Characteristic	Premenopausal, n = 601		Postmenopausal, n = 290	
	Nulliparous, n = 61	Parous, n = 540	Nulliparous, n = 21	Parous, n = 269
Mean (SD)				
% epithelium	8.8 (4.8)	10.4 (7.0)	4.6 (2.8)	7.2 (6.1)
% stroma	76.1 (10.3)	74.5 (11.0)	74.0 (10.7)	67.3 (12.6)
% fat	15.1 (11.2)	15.1 (10.9)	21.3 (9.9)	25.5 (13.5)
% fibroglandular tissue^a^	84.9 (11.2)	84.9 (10.9)	78.7 (9.9)	74.5 (13.5)
Age (years)^b^	36.2 (7.4)	41.5 (7.3)	55.1 (7.5)	57.3 (6.5)
Age at menopause (years)	NA	NA	44.5 (4.9)	48.4 (5.1)
Body mass index (kg/m^2^)	24.2 (5.4)	24.1 (4.5)	24.5 (2.4)	25.1 (4.0)
Alcohol use (g/day)	4.2 (4.7)	5.4 (8.8)	4.3 (3.8)	5.5 (8.6)
Parity	NA	2.7 (1.2)	NA	3.2 (1.5)
Age at first birth (years)	NA	25.1 (3.6)	NA	24.9 (3.2)
Age at menarche (years)	13.0 (1.1)	12.5 (1.4)	12.9 (0.8)	12.7(1.3)
Percentages^c^				
Breastfeeding				
0 to < 1 month	NA	41	NA	50
1 to < 12 months	NA	32	NA	37
12 to < 24 months	NA	18	NA	10
≥ 24 months	NA	10	NA	3
Family history of breast cancer	10	10	22	17
Smoking status				
Never smoked	61	56	24	49
Past smoker	20	28	45	34
Current smoker	20	16	31	17
Postmenopausal hormone use				
Never used	NA	NA	12	29
Past use	NA	NA	3	21
Current use	NA	NA	74	32
Unknown status	NA	NA	12	18
Benign breast disease subtypes				
Non-proliferative	23	32	39	27
Proliferative without atypia	70	58	45	55
Proliferative with atypia	7	10	15	18

*Note*: Values are means (SD) and percentages and are standardized to the age distribution of the study population

*Abbreviations*: *SD* standard deviation, *NA* not applicable

^a^Fibroglandular tissue represents combined epithelium and stroma

^b^Value is not age adjusted

^c^Percentages are calculated based on non-missing values

In multivariate analysis (Table 2), being nulliparous was significantly associated with a reduced proportion of epithelium (nulliparous vs. parous β = − 0.26, 95% confidence interval [CI] − 0.41, − 0.11) and fat tissue (nulliparous vs. parous β = − 0.34, 95% CI − 0.54, − 0.13) and an increased proportion of stroma (nulliparous vs. parous β = 0.04, 95% CI 0.01, 0.08). The duration of breastfeeding of 24 months or longer was associated with a reduced proportion of fat (breastfeeding ≥ 24 vs. 0 to < 1 month, β = − 0.30, 95% CI − 0.54, − 0.06; p-trend = 0.04). As during pregnancy and lactation, terminal duct-lobular units undergo differentiation with a simultaneous reduction in surrounding fat cells [36], we explored the potential effect of postpartum involution on the observed associations of breastfeeding with the proportion of fat by additionally adjusting these models for time since the last pregnancy. We observed a slight attenuation of the effects (β = − 0.22, 95% CI − 0.47; 0.03 with adjustment vs. β = − 0.30, 95% CI − 0.5; − 0.06 without adjustment). The findings also did not reach statistical significance (p-trend = 0.10), likely due to the smaller number of observations in these models (790 vs. 816) due to missing data on the time since the last pregnancy. Finally, we found no interaction of time since the last pregnancy with breastfeeding (p for interaction = 0.40).

**Table 2:** Associations of reproductive variables with the percentage of different tissue types (log-transformed) in benign breast biopsy samples (β coefficients and 95% confidence intervals)

Reproductive factor	Number	Tissue type			
		% epithelial	% stroma	% fat	% fibroglandular^a^
Nulliparity^b^					
Nulliparous	86	− 0.26 (− 0.41; − 0.11)	0.04 (0.01; 0.08)	− 0.34 (− 0.54; − 0.13)	0.01 (− 0.02; 0.05)
Parous	880	Ref	Ref	Ref	Ref
Breastfeeding, months^c^					
0 to < 1	361	Ref	Ref	Ref	Ref
1 to < 12	279	0.03 (− 0.07; 0.13)	0.02 (− 0.01; 0.04)	− 0.06 (− 0.18; 0.07)	0.01 (− 0.01; 0.04)
12 to < 24	119	0.08 (− 0.05; 0.22)	0.01 (− 0.03; 0.04)	− 0.05 (− 0.22; 0.12)	0.01 (− 0.03; 0.04)
≥ 24	57	0.04 (− 0.15; 0.23)	0.02 (− 0.03; 0.08)	− 0.30 (− 0.54; − 0.06)	0.03 (− 0.02; 0.07)
p-trend	816	0.39	0.43	0.04	0.34
Parity^d^					
1	82	Ref	Ref	Ref	Ref
2	292	0.11 (− 0.05; 0.27)	− 0.02 (− 0.06; 0.02)	− 0.05 (− 0.25; 0.15)	0.01 (− 0.03; 0.05)
3	269	0.12 (− 0.05; 0.28)	− 0.03 (− 0.08; 0.01)	0.01 (− 0.19; 0.22)	− 0.01(− 0.05; 0.03)
≥ 4	223	0.16 (− 0.02; 0.33)	− 0.05 (− 0.10; − 0.00)	0.04 (− 0.18; 0.26)	− 0.02(− 0.06; 0.03)
p-trend	866	0.13	0.02	0.32	0.17
Parity continuous^d^	866	0.03 (− 0.00; 0.07)	− 0.01 (− 0.02; − 0.00)	0.01 (− 0.03; 0.06)	− 0.01 (− 0.01; 0.00)
Age at first child’s birth^e^					
< 25	445	ref	ref	ref	ref
25–29	331	0.12 (0.03; 0.21)	− 0.01 (− 0.03; 0.02)	− 0.09 (− 0.20; 0.03)	0.01 (− 0.01; 0.03)
≥ 30	90	0.07 (− 0.08; 0.23)	− 0.03 (− 0.07; 0.01)	− 0.04 (− 0.23; 0.15)	− 0.01 (− 0.05; 0.03)
p-trend	866	0.13	0.16	0.44	0.85
Age at first birth continuous (per 5 years)^e^	866	0.04 (− 0.02; 0.11)	− 0.01 (− 0.02; 0.01)	− 0.03 (− 0.11; 0.05)	− 1.8 × 10^−3^ (− 0.01; 0.02)
Age at menarche^f^					
< 12	176	− 0.09 (− 0.22; 0.04)	− 0.01 (− 0.05; 0.02)	0.07 (− 0.12; 0.25)	− 0.01 (− 0.05; 0.02)
12	275	− 0.13 (− 0.24; − 0.01)	− 0.04 (− 0.07; − 0.01)	0.10 (− 0.06; 0.26)	− 0.05 (− 0.07; − 0.02)
13	287	− 0.06 (− 0.17; 0.05)	− 0.03 (− 0.06; − 0.00)	− 0.01 (− 0.17; 0.15)	− 0.03 (− 0.05; − 0.00)
> 13	228	ref	ref	ref	ref
p-trend	966	0.07	0.23	0.24	0.11
Age at menarche continuous (per 5 years)^f^	966	0.15 (− 0.01, 0.30)	0.02 (− 0.02, 0.06)	− 0.11 (− 0.33, 0.11)	0.03 (− 0.01, 0.06)
Time between menarche and age at first birth, continuous (per 5 years)^g^	866	0.02 (− 0.04; 0.08)	− 0.01 (− 0.02; 0.01)	− 0.01 (− 0.09; 0.06)	− 1.7 × 10^−3^ (− 0.02; 0.01)
Time since the last pregnancy, continuous (per 5 years)^c^	839	− 0.05 (− 0.12; 0.02)	− 0.02 (− 0.04 –3.0 × 10^−3^)	0.05 (− 0.03; 0.14)	− 0.02 (− 0.04; − 3.0 × 10^−3^)

^a^Fibroglandular tissue represents combined epithelium and stroma

^b^Adjusted for age (continuous), BMI (continuous), age at menarche (< 12, 12, 13, > 13), a family history of breast cancer (yes/no), menopausal status/postmenopausal hormone use (premenopausal, postmenopausal/no hormones, postmenopausal/past hormones, postmenopausal/current hormones, postmenopausal/unknown hormone use status), NHS cohort (NHSI, NHSII), and alcohol use (none, > 0 to < 5, ≥ 5 g/day)

^c^Among parous women only: adjusted for age (continuous), BMI (continuous), age at menarche (< 12, 12, 13, > 13), parity, age at first child’s birth, a family history of breast cancer (yes/no), menopausal status/postmenopausal hormone use (premenopausal, postmenopausal/no hormones, postmenopausal/past hormones, postmenopausal/current hormones, postmenopausal/unknown hormone use status), NHS cohort (NHSI, NHSII), and alcohol use (none, > 0 to < 5, ≥5 g/day)

^d^Among parous women only: adjusted for age (continuous), BMI (continuous), age at first birth, age at menarche (< 12, 12, 13, > 13), a family history of breast cancer (yes/no), menopausal status/postmenopausal hormone use (premenopausal, postmenopausal/no hormones, postmenopausal/past hormones, postmenopausal/current hormones, postmenopausal/unknown hormone use status), NHS cohort (NHSI, NHSII), and alcohol use (none, > 0 to < 5, ≥5 g/day)

^e^Among parous women only: adjusted for age (continuous), BMI (continuous), parity, age at menarche (< 12, 12, 13, > 13), a family history of breast cancer (yes/no), menopausal status/postmenopausal hormone use (premenopausal, postmenopausal/no hormones, postmenopausal/past hormones, postmenopausal/current hormones, postmenopausal/unknown hormone use status), NHS cohort (NHSI, NHSII), and alcohol use (none, > 0 to < 5, ≥5 g/day)

^f^Adjusted for age (continuous), BMI (continuous), parous status (nulliparous, parous), a family history of breast cancer (yes/no), menopausal status/postmenopausal hormone use (premenopausal, postmenopausal/no hormones, postmenopausal/past hormones, postmenopausal/current hormones, postmenopausal/unknown hormone use status), NHS cohort (NHSI, NHSII), and alcohol use (none, > 0 to < 5, ≥5 g/day)

^g^Among parous women only: adjusted for age (continuous), BMI (continuous), parity, a family history of breast cancer (yes/no), menopausal status/postmenopausal hormone use (premenopausal, postmenopausal/no hormones, postmenopausal/past hormones, postmenopausal/current hormones, postmenopausal/unknown hormone use status), NHS cohort (NHSI, NHSII), and alcohol use (none, > 0 to < 5, ≥ 5 g/day)

Parity was associated with a reduced proportion of stroma (β per one child = − 0.01, 95% CI − 0.02, − 0.00; p-trend 0.02), and having a first child at age 25–29 years was associated with a larger proportion of epithelial tissue (age at first birth 25–29 vs. < 25 years, β = 0.12, 95% CI 0.03, 0.21), though there was no clear pattern for this association. The time since the last pregnancy as well as the duration of the interval between age at menarche and first birth was not associated with any of the tissue measures. None of the reproductive factors was associated with the proportion of fibroglandular tissue. The patterns of associations of the reproductive factors with tissue types were similar in the statistical analyses with the secondary modeling approach (Supplementary Table 2).

Among premenopausal women (Table 3), being nulliparous was associated with a greater proportion of stroma (nulliparous vs. parous β = 0.06, 95% CI 0.02, 0.10) and a smaller proportion of epithelium (nulliparous vs. parous β = − 0.22, 95% CI − 0.38, − 0.06) and fat (nulliparous vs. parous β = − 0.32, 95% CI − 0.56, − 0.08). Greater parity and older age at first birth were both associated with a greater proportion of epithelium and a smaller proportion of stroma. The time since the last pregnancy as well as the duration of the interval between age at menarche and first birth was not associated with the proportion of any of the tissue types. These patterns of associations were similar with the secondary modeling approach (Supplementary Table 3). Finally, the findings did not change after additional adjustment for the BBD subtype (Supplementary tables 4 and 5).

**Table 3:** Associations of reproductive variables with the percentage of different tissue types (log-transformed) in benign breast biopsy samples of premenopausal women (β coefficients and 95% confidence intervals)

Reproductive factor	Number	Tissue type			
		% epithelial	% stroma	% fat	% fibroglandular^a^
Nulliparity^b^					
Nulliparous	61	− 0.22 (− 0.38; − 0.06)	0.06 (0.02; 0.10)	− 0.32 (− 0.56; − 0.08)	0.03 (− 0.01; 0.06)
Parous	540	Ref	Ref	Ref	Ref
Breastfeeding, months^c^					
0 to < 1	210	Ref	Ref	Ref	Ref
1 to < 12	162	− 0.06 (− 0.18; 0.06)	0.04 (0.01; 0.07)	− 0.11 (− 0.28; 0.06)	0.03 (− 0.00; 0.05)
12 to < 24	92	− 0.02 (− 0.17; 0.13)	0.01 (− 0.03; 0.06)	− 0.02 (− 0.24; 0.19)	0.00 (− 0.03; 0.04)
≥ 24	47	− 0.09 (− 0.30; 0.11)	0.04 (− 0.02; 0.09)	− 0.18 (− 0.48; 0.11)	0.02 (− 0.02; 0.07)
p-trend	511	0.53	0.33	0.39	0.57
Parity^d^					
1	62	Ref	Ref	Ref	Ref
2	190	0.27 (0.10; 0.44)	− 0.02 ( − 0.07; 0.03)	− 0.17 (− 0.42; 0.07)	0.02 (− 0.02; 0.06)
3	167	0.25 (0.08; 0.43)	− 0.04 (− 0.09; 0.01)	− 0.03 (− 0.29; 0.22)	− 0.00 (− 0.04; 0.04)
≥ 4	108	0.34 (0.14; 0.53)	− 0.06 (− 0.11; − 0.00)	0.03 (− 0.25; 0.30)	− 0.01 (− 0.05; 0.04)
p-trend	527	0.01	0.02	0.25	0.23
Parity continuous^d^	527	0.07 (0.02; 0.11)	− 0.02 (− 0.03; − 0.00)	0.03 (− 0.03; 0.10)	− 0.01 (− 0.02; 0.00)
Age at first child’s birth^e^					
< 25	263	Ref	Ref	Ref	Ref
25–29	205	0.20 (0.10; 0.31)	− 0.01 (− 0.04; 0.02)	− 0.19 (− 0.34; − 0.03)	0.02 (− 0.01; 0.04)
≥ 30	59	0.23 (0.06; 0.40)	− 0.05 (− 0.10; − 0.00)	0.01 (− 0.24; 0.26)	− 0.01 (− 0.05; 0.03)
p-trend	527	< 0.01	0.05	0.58	0.99
Age at first birth continuous (per 5 years)^e^	527	0.10 (0.03; 0.17)	− 0.01 (− 0.03; 0.01)	− 0.04 (− 0.14; 0.06)	4.5 × 10^−3^ (− 0.01; 0.02)
Age at menarche^f^					
< 12	115	− 0.10 (− 0.25; 0.06)	− 0.01 (− 0.05; 0.03)	0.10 (− 0.13; 0.33)	− 0.02 (− 0.05; 0.02)
12	174	− 0.14 (− 0.28; − 0.00)	− 0.03 (− 0.06; 0.01)	0.07 (− 0.14; 0.28)	− 0.03 (− 0.07; − 0.00)
13	187	− 0.01 (− 0.14; 0.13)	− 0.02 (− 0.05; 0.02)	− 0.02 (− 0.22; 0.18)	− 0.01 (− 0.04; 0.02)
> 13	125	Ref	Ref	Ref	Ref
p-trend	601	0.06	0.61	0.24	0.14
Age at menarche continuous (per 5 years)^f^	601	0.16 (− 0.02, 0.33)	0.01 (− 0.04, 0.05)	− 0.13 (− 0.40, 0.13)	0.03 (− 0.02, 0.07)
Time between menarche and age at first birth, continuous (per 5 years)^g^	527	0.07 (− 8.0 × 10^−3^; 0.14)	− 0.01 (− 0.03; 0.01)	− 0.01 (− 0.11; 0.09)	− 9.0 × 10^−3^ (− 0.02; 0.02)
Time since the last pregnancy, continuous (per 5 years)^c^	501	− 0.04 (− 0.12; 0.05)	− 0.02 (− 0.04; 3.8 × 10^−3^)	0.07 (− 0.04; 0.19)	− 0.02 (− 0.04; 2.1 × 10^−3^)

^a^Fibroglandular tissue represents combined epithelium and stroma

^b^Adjusted for age (continuous), BMI (continuous), age at menarche (< 12, 12, 13, > 13), a family history of breast cancer (yes/no), NHS cohort (NHSI, NHSII), and alcohol use (none, > 0 to < 5, ≥5 g/day)

^c^Among parous women only: adjusted for age (continuous), BMI (continuous), age at menarche (< 12, 12, 13, > 13), parity (1, 2, 3, and ≥4 children), age at first child’s birth (<25, 25-29, and ≥30 years), a family history of breast cancer (yes/no), NHS cohort (NHSI, NHSII), and alcohol use (none, > 0 to < 5, ≥5 g/day)

^d^Among parous women only: adjusted for age (continuous), BMI (continuous), age at first birth (<25, 25-29, and ≥30 years), age at menarche (< 12, 12, 13, > 13), a family history of breast cancer (yes/no), NHS cohort (NHSI, NHSII), and alcohol use (none, > 0 to < 5, ≥5 g/day)

^e^Among parous women only: adjusted for age (continuous), BMI (continuous), parity (1, 2, 3, and ≥4 children), age at menarche (< 12, 12, 13, > 13), a family history of breast cancer (yes/no), NHS cohort (NHSI, NHSII), and alcohol use (none, > 0 to < 5, ≥5 g/day)

^f^Adjusted for age (continuous), BMI (continuous), parous status (nulliparous, parous), a family history of breast cancer (yes/no), NHS cohort (NHSI, NHSII), and alcohol use (none, > 0 to < 5, ≥5 g/day)

^g^Among parous women only: adjusted for age (continuous), BMI (continuous), parity (continuous), a family history of breast cancer (yes/no), NHS cohort (NHSI, NHSII), and alcohol use (none, > 0 to < 5, ≥5 g/day)

## Discussion

In this study of 983 cancer-free women, being nulliparous was associated with having a smaller percentage of epithelium and fat and a greater percentage of stroma, while the greater number of children was associated with a smaller percentage of stroma. Breastfeeding for 24 months or longer was associated with a decreased percentage of fat. No associations were observed for the interval between menarche and age at first birth and the positive associations of age at first birth with the percentage of epithelium and inverse associations with the percentage of stroma were seen in premenopausal women.

Consistent with our findings on the associations of nulliparity with epithelium, a recent study by Gabrielson et al. of core biopsy samples of non-malignant breast tissue from 153 cancer-free women found a greater proportion of epithelium in parous as compared to nulliparous women (parous vs. nulliparous β = 0.56, p = 0.07) [20]. In contrast, an earlier study by Gertig et al. within NHS found no associations of parity with the proportion of epithelium or stroma. Compared to our study, this study was small (n = 300) and used a different method of computer-assisted image analysis [21]. Finally, even though neither of the previous studies found associations of parity with the proportion of stroma, Gabrielson et al. observed inverse associations with stromal proliferation [20].

Breast tissue changes during pregnancy have been suggested as possible reasons for the long-term protective effect on breast cancer risk [37]. The influence of full-term pregnancy on the breast tissue appears to be complex and some of the suggested mechanisms include changes in hormonal signaling in the breast, gene methylation and expression changes, long-term reduction in the levels of circulating hormones, and life-long reduction in the number of mammary stem cells [4, 10, 38–40]. Previous studies also suggest that hormonal changes during pregnancy may also influence the stromal composition, but the evidence remains inconsistent [16, 41, 42]. Interestingly, in nulliparous women in our study, we observed a smaller proportion of epithelium but a larger proportion of stroma. These findings suggest that the increased risk of breast cancer in nulliparous women might potentially be driven by the dominating stroma and epithelial-stromal interactions that play a pivotal role in normal mammary gland function by controlling and regulating normal processes in the breast and suppressing the expression of preneoplastic phenotypes [43, 44]. Further, despite these findings for stroma and epithelium, we did not observe associations with the percentage of fibroglandular tissue which could further indicate that the relative contributions of stroma and epithelium might be more important than their absolute amount. Importantly, in our previous investigation of associations of reproductive factors with breast density, we observed greater percent breast density in nulliparous as compared to parous women. A few previous studies suggested that the degree of mammographic breast density is driven predominantly by the changes in the extent and composition of stroma rather than epithelium [45–47]. Thus, these relationships appear complex and warrant further investigation. Finally, as previous studies show heterogeneity in the association of reproductive factors with molecular breast cancer subtypes, our findings might help to explain eventually the underlying mechanisms behind these associations. However, as our study did not look at the associations with breast cancer outcomes and was limited to cancer-free women only, we are unable to discuss this further in the context of our findings.

In our study, older age at first birth was associated with a greater proportion of epithelium and a smaller proportion of stroma, but these findings were limited to premenopausal women. We did not find any associations of the length of the time period between menarche and first birth with any of the tissue types. Gabrielson et al. found a marginally positive association of age at first birth with the proportion of epithelium, but the results were not significant in the small stratum (n = 55) of premenopausal women [20], and Gertig et al. did not find any associations [21]. Younger age at first birth may reduce subsequent breast cancer risk by earlier induction of cellular differentiation in the breast [48]. However, it remains unclear if the observed associations may represent the result of the long-lasting effects of this differentiation.

We report inverse associations of breastfeeding duration with the proportion of fat tissue. Consistent with our findings, Gabrielson et al. found inverse associations, though only marginally significant associations of breastfeeding with the percentage of adipose tissue in the breast (β = − 0.55, p = 0.05) [20]. Studies on breast tissue remodeling after lactation in humans are very limited though some studies suggest that the protective effect of breastfeeding on breast cancer risk may be related to the increased cellular proliferation and epithelial exfoliation of breast tissue during lactation with subsequent apoptosis after discontinuation of breastfeeding that could result in the elimination of cells which may have DNA damage [4, 10, 38–40]. Animal models suggest that with discontinuation of lactation, the breast tissue undergoes postpartum involution and remodeling as the result of apoptosis, regression of alveoli, and adipocyte repopulation [36, 49]. During lactation, mammary gland adipose tissue undergoes significant remodeling with replacement of adipocytes by mammary alveolar structures and their subsequent re-differentiation (“reversion”) back into adipocytes after weaning [50]. Whether this reversion completely restores the previous tissue structure and whether this mechanism could be applied to humans is unknown. Finally, some recent studies also suggest that women who breastfed may have lower adiposity as reflected in their BMI which could also affect the amount of adipose tissue in the breast [51]. In our study, however, we observed no correlation between BMI and breastfeeding (correlation coefficient = − 0.01, p = 0.75), and the estimates for the duration of breastfeeding in all models were adjusted for BMI.

To our knowledge, this is the largest study to date exploring the associations of several reproductive variables with the proportion of epithelium, stroma, fibroglandular, and fat tissues. The analysis used data from the Nurses’ Health Study and Nurses’ Health Study II, established cohorts with more than 30 years of follow-up, confirmed benign breast disease status and comprehensive information on breast cancer risk factors. Our study has a few limitations. Despite the prospective nature of the cohort, a measurement error for a few select reproductive variables especially in postmenopausal women is possible. For example, previous studies had conflicting findings on the accuracy of recall for age at menarche [52–54] which could potentially influence the results for associations of the interval between menarche and first birth with tissue measures. Some reports suggest that recall inaccuracy for breastfeeding in older women can affect the estimated associations between breastfeeding and health outcomes [55]. Next, the proportion of nulliparous women in our study (10%) is comparable to the proportion among cancer-free women from other studies (range 4–14% [56–59]), including those in NHS/NHSII [60, 61]. As our study includes only cancer-free women with clinically indicated biopsy resulting in BBD diagnosis and since the analysis of the whole-slide images included both the background normal tissue as well as benign lesions, the findings are expected to be generalizable to cancer-free women with BBD. Next, as whole-slide images included both normal tissue and lesions, we additionally adjusted our models for BBD subtypes to explore their influence on the study findings, and the results remained unchanged. Finally, due to the small proportion of postmenopausal women in our study sample (n = 290), we were unable to perform an analysis within this stratum.

## Conclusions

We investigated the associations of several reproductive variables related to childbearing with the extent of epithelial, stromal, fibroglandular, and fat tissue. Our findings suggest that nulliparous women are more likely to have a lower percentage of epithelium and fat and a greater percentage of stroma as compared to parous women, potentially suggesting the importance of epithelial-stromal interactions. Parous women with a greater number of children appeared to have a smaller proportion of stroma. In premenopausal women, younger age at first birth was associated with a larger proportion of epithelium and a smaller proportion of stroma. Future studies are warranted to confirm our findings and to elucidate the underlying biological mechanisms.

## Supplementary Information

**Additional file 1: Table S1.** Distribution of various tissue elements by the type of benign breast disease. **Table S2.** Associations of reproductive factors with tissue types in benign breast biopsy samples using proc glimmix procedure (Odds Ratios and 95% Confidence Intervals). **Table S3.** Associations of reproductive variables with tissue types in benign breast biopsy samples of premenopausal women using proc glimmix procedure (Odds Ratios and 95% Confidence Intervals). **Table S4.** Associations of reproductive variables with percentage of different tissue types (log-transformed) in benign breast biopsy samples (β coefficients and 95% Confidence Intervals), additionally adjusted for BBD subtype. **Table S5.** Associations of reproductive variables with percentage of different tissue types (log-transformed) in benign breast biopsy samples of premenopausal women (β coefficients and 95% Confidence Intervals), additionally adjusted for BBD subtype.

## Data Availability

Not applicable.

## Abbreviations

BBD: Benign breast disease
BMI: Body mass index
CI: Confidence interval
H&E: Hematoxylin and eosin
NHS: Nurses’ Health Study
OR: Odds ratio
