# Supplementary material for: Associations of reproductive breast cancer risk factors with breast tissue composition
Source: Breast Cancer Res. 2021 Jul 5;23:70. doi: 10.1186/s13058-021-01447-2 (PMC8258947; doi:10.1186/s13058-021-01447-2)
Supplement: Supplementary file 1 — Additional file 1: Table S1. Distribution of various tissue elements by the type of benign breast disease. Table S2. Associations of reproductive factors with tissue types in benign breast biopsy samples using proc glimmix procedure (Odds Ratios and 95% Confidence Intervals). Table S3. Associations of reproductive variables with tissue types in benign breast biopsy samples of premenopausal women using proc glimmix procedure (Odds Ratios and 95% Confidence Intervals). Table S4. Associations of reproductive variables with percentage of different tissue types (log-transformed) in benign breast biopsy samples (β coefficients and 95% Confidence Intervals), additionally adjusted for BBD subtype. Table S5. Associations of reproductive variables with percentage of different tissue types (log-transformed) in benign breast biopsy samples of premenopausal women (β coefficients and 95% Confidence Intervals), additionally adjusted for BBD subtype. [file 13058_2021_1447_MOESM1_ESM.docx]

**Supplementary Table 1. Distribution of various tissue elements by the type of benign breast disease**

|  | **All women** |  |  | **Premenopausal** | | | **Postmenopausal** | | |
| --- | --- | --- | --- | --- | --- | --- | --- | --- | --- |
| **Tissue type** | **Non-proliferative (n=299)** | **Proliferative without atypia (n=559)** | **Proliferative with atypia (n=125)** | **Non-proliferative (n=191)** | **Proliferative without atypia**  **(n= 358)** | **Proliferative with atypia (n= 63)** | **Non-**  **proliferative (n=81 )** | **Proliferative without atypia**  **(n= 160)** | **Proliferative with atypia (n= 54)** |
| **% Epithelium**  Mean (SD)  Range | 8.0 (6.8)  0.5-49.3 | 9.6 (6.7)  1.0-52.2 | 9.0 (5.7)  0.7-40.8 | 9.6 (7.0)  0.8-49.3 | 10.5 (6.9)  2.1-52.2 | 9.4 (4.9)  2.4-22.2 | 5.4 (6.0)  0.5-40.4 | 7.5 (5.5)  1.0-40.3 | 8.5 (6.7)  0.7-40.8 |
| **% Stroma**  Mean (SD)  Range | 71.1 (14.0)  23.6-99.0 | 73.1 (11.3)  29.6-96.7 | 72.8 (10.4)  41.5-93.2 | 73.8 (13.0)  23.6-98.4 | 75.8 (10.2)  32.7-96.7 | 75.1 (9.7)  49.0-93.2 | 65.1 (14.8)  33.3-99.0 | 67.7 (12.1)  29.6-94.1 | 71.3 (10.7)  41.5-91.8 |
| **% Fat**  Mean (SD)  Range | 20.9 (14.7)  0-71.3 | 17.3 (12.1)  0-65.8 | 18.3 (11.0)  1.6-56.5 | 16.6 (12.8)  0.3-71.3 | 13.7 (10.3)  0-60.0 | 15.5 (9.6)  1.6-45.0 | 29.5 (14.8)  0.0-65.4 | 24.9 (12.8)  0.1-65.8 | 20.2 (11.8)  2.7-56.5 |
| **% Fibroglandular**  Mean (SD)  Range | 79.1 (14.7)  28.7-100 | 82.7 (12.1)  34.2-100 | 81.7 (11.0)  43.5-98.4 | 83.4 (12.8)  28.7-99.7 | 86.3 (10.3)  40.0-100 | 84.5 (9.6)  55.0-98.4 | 70.5 (14.8)  34.6-100 | 75.1 (12.8)  34.2-99.9 | 79.8 (11.8)  43.5-97.3 |

**Supplementary Table 2. Associations of reproductive factors with tissue types in benign breast biopsy samples using proc glimmix procedure (Odds Ratios and 95% Confidence Intervals)**

| **Reproductive factor** | **N** | **Tissue type** | | | |
| --- | --- | --- | --- | --- | --- |
|  |  | **% Epithelial** | **% Stroma** | **% Fat** | **% Fibroglandular ^a^** |
| **Nulliparity^b^**  **Nulliparous**  **Parous** | 86  880 | 0.77 (0.64; 0.93)  ref | 1.19 (1.04; 1.37)  ref | 0.91 (0.75; 1.09)  ref | 1.09 (0.90; 1.31)  ref |
| **Breastfeeding, months^c^**  **0-<1**  **1-<12**  **12-<24**  **≥24**  **p-trend** | 361  279  119  57  816 | ref  0.99 (0.88; 1.12)  1.01 (0.86; 1.18)  1.05 (0.84; 1.30)  0.71 | ref  1.05 (0.96; 1.15)  1.02 (0.91; 1.15)  1.08 (0.91; 1.29)  0.43 | ref  0.94 (0.84; 1.06)  0.97 (0.82; 1.15)  0.86 (0.67; 1.09)  0.28 | ref  1.06 (0.94; 1.19)  1.03 (0.87; 1.21)  1.17 (0.92; 1.49)  0.28 |
| **Parity ^d^**  **1**  **2**  **3**  **≥4**  **p-trend** | 82  292  269  223  866 | ref  1.26 (1.03; 1.53)  1.21 (0.99; 1.49)  1.31 (1.06; 1.62)  0.09 | ref  0.93 (0.80; 1.07)  0.90 (0.77; 1.04)  0.85 (0.72; 0.99)  0.02 | ref  0.98 (0.81; 1.20)  1.05 (0.85; 1.28)  1.09 (0.88; 1.34)  0.19 | ref  1.02 (0.84; 1.24)  0.96 (0.78; 1.17)  0.92 (0.75; 1.14) 0.19 |
| **Parity continuous ^d^** | 866 | 1.04 (1.00; 1.08) | 0.97 (0.94; 1.00) | 1.03 (0.99; 1.07) | 0.97 (0.94; 1.01) |
| **Age at first child’s birth ^e^**  **<25**  **25-29**  **≥30**  **p-trend** | 445  331  90  866 | ref 1.14 (1.02; 1.27)  1.19 (0.99; 1.43)  0.02 | ref  0.97 (0.90; 1.05)  0.91 (0.80; 1.04)  0.16 | ref  0.96 (0.86; 1.07)  1.03 (0.85; 1.23)  0.98 | ref  1.04 (0.93; 1.16)  0.98 (0.81; 1.17)  0.98 |
| **Age at first birth continuous (years) ^e^** | 866 | 1.01 (1.00; 1.03) | 1.00 (0.99; 1.01) | 1.00 (0.98; 1.01) | 1.00 (0.99; 1.02) |
| **Age at menarche ^f^**  **<12**  **12**  **13**  **>13**  **p-trend** | 176  275  287  228  966 | 0.96 (0.83, 1.13)  0.91 (0.79, 1.04)  1.01 (0.88, 1.15)  ref  0.28 | 0.96 (0.86, 1.07)  0.89 (0.81, 0.98)  0.91 (0.83, 1.01)  ref  0.29 | 1.08 (0.93, 1.20)  1.23 (1.07, 1.41) 1.12 (0.98, 1.29)  ref  0.11 | 0.93 (0.79, 1.08)  0.81 (0.71, 0.93)  0.89 (0.77, 1.02)  ref  0.11 |
| **Age at menarche continuous (years) ^f^** | 966 | 1.02 (0.99, 1.06) | 1.01 (0.98, 1.04) | 0.97 (0.94, 1.01) | 1.03 (0.99, 1.07) |
| **Time between menarche and age at first birth, continuous (years) ^g^** | 866 | 1.01 (1.00; 1.02) | 1.00 (0.99; 1.01) | 1.00 (0.99; 1.02) | 1.00 (0.98; 1.01) |
| **Time since last pregnancy, continuous (years) ^c^** | 839 | 0.99 (0.98; 1.01) | 0.99 (0.98; 1.00) | 1.02 (1.00; 1.03) | 0.98 (0.97; 1.00) |

^a^ Fibroglandular tissue represents combined epithelium and stroma

**^b^** Adjusted for age (continuous), BMI (continuous), age at menarche (<12, 12, 13, >13), a family history of breast cancer (Yes/No), menopausal status/postmenopausal hormone use (premenopausal, postmenopausal/no hormones, postmenopausal/past hormones, postmenopausal/current hormones, postmenopausal/unknown hormone use status), NHS cohort (NHSI, NHSII), and alcohol use (none, >0-<5, ≥5 g/day)

**^c^** Among parous women only: adjusted for age (continuous), BMI (continuous), age at menarche (<12, 12, 13, >13), parity, age at first child’s birth, a family history of breast cancer (Yes/No), menopausal status/postmenopausal hormone use (premenopausal, postmenopausal/no hormones, postmenopausal/past hormones, postmenopausal/current hormones, postmenopausal/unknown hormone use status), NHS cohort (NHSI, NHSII), and alcohol use (none, >0-<5, ≥5 g/day)

**^d^** Among parous women only: adjusted for age (continuous), BMI (continuous), age at first birth, age at menarche (<12, 12, 13, >13), a family history of breast cancer (Yes/No), menopausal status/postmenopausal hormone use (premenopausal, postmenopausal/no hormones, postmenopausal/past hormones, postmenopausal/current hormones, postmenopausal/unknown hormone use status), NHS cohort (NHSI, NHSII), and alcohol use (none, >0-<5, ≥5 g/day)

**^e^** Among parous women only: adjusted for age (continuous), BMI (continuous), parity, age at menarche (<12, 12, 13, >13), a family history of breast cancer (Yes/No), menopausal status/postmenopausal hormone use (premenopausal, postmenopausal/no hormones, postmenopausal/past hormones, postmenopausal/current hormones, postmenopausal/unknown hormone use status), NHS cohort (NHSI, NHSII), and alcohol use (none, >0-<5, ≥5 g/day)

**^f^** Adjusted for age (continuous), BMI (continuous), parous status (nulliparous, parous), a family history of breast cancer (Yes/No), menopausal status/postmenopausal hormone use (premenopausal, postmenopausal/no hormones, postmenopausal/past hormones, postmenopausal/current hormones, postmenopausal/unknown hormone use status), NHS cohort (NHSI, NHSII), and alcohol use (none, >0-<5, ≥5 g/day)

**^g^** Among parous women only: adjusted for age (continuous), BMI (continuous), parity, a family history of breast cancer (Yes/No), menopausal status/postmenopausal hormone use (premenopausal, postmenopausal/no hormones, postmenopausal/past hormones, postmenopausal/current hormones, postmenopausal/unknown hormone use status), NHS cohort (NHSI, NHSII), and alcohol use (none, >0-<5, ≥5 g/day)

**Supplementary Table 3. Associations of reproductive variables with tissue types in benign breast biopsy samples of premenopausal women using proc glimmix procedure (Odds Ratios and 95% Confidence Intervals)**

| **Reproductive variable** | **N** | **Tissue type** | | | |
| --- | --- | --- | --- | --- | --- |
|  |  | **% Epithelial** | **% Stroma** | **% Fat** | **% Fibroglandular^a^** |
| **Nulliparity^b^**  **Nulliparous**  **Parous** | 61  540 | 0.78 (0.64; 0.96)  ref | 1.28 (1.09; 1.52)  ref | 0.82 (0.64; 1.04)  ref | 1.20 (0.94; 1.53)  ref |
| **Breastfeeding, months^c^**  **0-<1**  **1-<12**  **12-<24**  **≥24**  **p-trend** | 210  162  92  47  511 | ref  0.93 (0.81; 1.08)  0.92 (0.77; 1.10)  0.93 (0.74; 1.18)  0.45 | ref  1.14 (1.01; 1.28)  1.05 (0.91; 1.21)  1.13 (0.92; 1.38)  0.38 | ref  0.87 (0.73; 1.03)  0.99 (0.81; 1.22)  0.87 (0.65; 1.16)  0.60 | ref  1.15 (0.97; 1.36)  1.01 (0.82; 1.24)  1.15 (0.86; 1.54)  0.60 |
| **Parity ^d^**  **1**  **2**  **3**  **≥4**  **p-trend** | 62  190  167  108  527 | ref  1.47 (1.19; 1.82) 1.37 (1.09; 1.71) 1.51 (1.19; 1.92)  0.03 | ref  0.92 (0.78; 1.09)  0.87 (0.73; 1.04)  0.81 (0.67; 0.98)  0.02 | ref  0.87 (0.69; 1.10)  1.02 (0.80; 1.30)  1.05 (0.81; 1.36)  0.20 | ref  1.15 (0.91; 1.45) 0.98 (0.77; 1.26)  0.95 (0.73; 1.24)  0.20 |
| **Parity continuous ^d^** | 527 | 1.06 (1.01; 1.12) | 0.94 (0.90; 0.98) | 1.04 (0.98; 1.11) | 0.96 (0.90; 1.02) |
| **Age at first child’s birth ^e^**  **<25**  **25-29**  **≥30**  **p-trend** | 263  205  59  527 | ref  1.27 (1.12; 1.43)  1.38 (1.13; 1.69)  <0.01 | ref  0.96 (0.86; 1.06)  0.83 (0.70; 0.98)  0.03 | ref  0.91 (0.78; 1.06)  1.05 (0.83; 1.33)  0.98 | ref  1.10 (0.95; 1.28)  0.96 (0.76; 1.21)  0.98 |
| **Age at first birth**  **Continuous (years) ^e^** | 527 | 1.03 (1.01; 1.04) | 0.99 (0.98; 1.00) | 1.00 (0.98; 1.02) | 1.01 (0.99; 1.03) |
| **Age at menarche ^f^**  **<12**  **12**  **13**  **>13**  **p-trend** | 115  174  187  125  601 | 0.92 (0.77,1.11)  0.90 (0.76,1.06)  1.05 (0.90,1.23)  ref  0.11 | 0.97 (0.84,1.13)  0.93 (0.82,1.07)  0.94 (0.83,1.08)  ref  0.68 | 1.12 (0.90, 1.38)  1.21 (1.00, 1.47)  1.06 (0.87, 1.28)  ref  0.13 | 0.89 (0.72, 1.10)  0.82 (0.68, 1.00)  0.94 (0.78, 1.14)  ref  0.13 |
| **Age at menarche continuous (years) ^f^** | 601 | 1.03 (0.99, 1.08) | 1.00 (0.97, 1.04) | 0.97 (0.92, 1.02) | 1.03 (0.98, 1.08) |
| **Time between menarche and age at first birth, continuous (years) ^g^** | 527 | 1.02 (1.00; 1.04) | 0.99 (0.98; 1.00) | 1.00 (0.98; 1.02) | 1.00 (0.98; 1.02) |
| **Time since last pregnancy, continuous (years) ^c^** | 501 | 0.99 (0.97; 1.01) | 0.99 (0.97; 1.01) | 1.02 (1.00; 1.05) | 0.98 (0.96; 1.00) |

^a^ Fibroglandular tissue represents combined epithelium and stroma

**^b^** Adjusted for age (continuous), BMI (continuous), age at menarche (<12, 12, 13, >13), a family history of breast cancer (Yes/No), NHS cohort (NHSI, NHSII), and alcohol use (none, >0-<5, ≥5 g/day)

**^c^** Among parous women only: adjusted for age (continuous), BMI (continuous), age at menarche (<12, 12, 13, >13), parity, age at first child’s birth, a family history of breast cancer (Yes/No), NHS cohort (NHSI, NHSII), and alcohol use (none, >0-<5, ≥5 g/day)

**^d^** Among parous women only: adjusted for age (continuous), BMI (continuous), age at first birth, age at menarche (<12, 12, 13, >13), a family history of breast cancer (Yes/No), NHS cohort (NHSI, NHSII), and alcohol use (none, >0-<5, ≥5 g/day)

**^e^** Among parous women only: adjusted for age (continuous), BMI (continuous), parity, age at menarche (<12, 12, 13, >13), a family history of breast cancer (Yes/No), NHS cohort (NHSI, NHSII), and alcohol use (none, >0-<5, ≥5 g/day)

**^f^** Adjusted for age (continuous), BMI (continuous), parous status (nulliparous, parous), a family history of breast cancer (Yes/No), NHS cohort (NHSI, NHSII), and alcohol use (none, >0-<5, ≥5 g/day)

^g^ Among parous women only: adjusted for age (continuous), BMI (continuous), parity, a family history of breast cancer (Yes/No), NHS cohort (NHSI, NHSII), and alcohol use (none, >0-<5, ≥5 g/day)

**Supplementary table 4.** **Associations of reproductive variables with percentage of different tissue types (log-transformed) in benign breast biopsy samples (β coefficients and 95% Confidence Intervals), additionally adjusted for BBD subtype.**

| **Reproductive factor** | **N** | **Tissue type** | | | | |
| --- | --- | --- | --- | --- | --- | --- |
|  |  | **% Epithelial** | **% Stroma** | **% Fat** | **% Fibroglandular^a^** |  |
| **Nulliparity^b^**  **Nulliparous**  **Parous** | 86  880 | -0.27 (-0.41; -0.12)  ref | 0.04 (0.00; 0.08)  ref | -0.33 (-0.54; -0.12) ref | 0.01 (-0.02; 0.05)  ref |  |
| **Breastfeeding, months^c^**  **0-<1**  **1-<12**  **12-<24**  **≥24**  **p-trend** | 361  279  119  57  816 | ref  0.02 (-0.08; 0.11)  0.07 (-0.06; 0.20)  0.02 (-0.16; 0.21)  0.48 | ref  0.01 (-0.01; 0.04)  0.01 (-0.03; 0.04)  0.02 (-0.03; 0.07)  0.48 | ref  -0.05 (-0.17; 0.08)  -0.04 (-0.21; 0.13)  -0.29 (-0.53; -0.05)  0.05 | ref  0.01 (-0.01; 0.03)  0.00 (-0.03; 0.04)  0.02 (-0.02; 0.07)  0.41 |  |
| **Parity ^d^**  **1**  **2**  **3**  **≥4**  **p-trend** | 82  292  269  223  866 | ref  0.13 (-0.03; 0.28)  0.13 (-0.03; 0.29)  0.19 (0.02; 0.36)  0.06 | ref  -0.02 (-0.06; 0.02)  -0.03 (-0.08; 0.01)  -0.04 (-0.09; 0.00)  0.04 | ref  -0.07 (-0.27; 0.13)  0.00 (-0.21; 0.21)  0.02 (-0.20; 0.23)  0.42 | ref  0.01 (-0.03; 0.05)  -0.01 (-0.05; 0.03)  -0.01 (-0.05; 0.03)  0.26 |  |
| **Parity continuous ^d^** | 866 | 0.04 (0.00; 0.07) | -0.01 (-0.02; 0.00) | 0.01 (-0.03; 0.05) | -0.01 (-0.01; 0.00) |  |
| **Age at first child’s birth ^e^**  **<25**  **25-29**  **≥30**  **p-trend** | 445  331  90  866 | ref  0.11 (0.02; 0.20)  0.10 (-0.05; 0.25)  0.06 | ref  -0.01 (-0.03; 0.02)  -0.03 (-0.07; 0.02)  0.19 | ref  -0.08 (-0.20; 0.03)  -0.06 (-0.26; 0.13)  0.33 | ref  0.01 (-0.02; 0.03)  -0.00 (-0.04; 0.03)  0.96 |  |
| **Age at first birth continuous (per 5 years) ^e^** | 866 | 0.05 (-0.01; 0.11) | -0.01 (-0.02; 0.01) | -0.04 (-0.12; 0.04) | -2.9x10^-3^ (-0.01; 0.02) |  |
| **Age at menarche ^f^**  **<12**  **12**  **13**  **>13**  **p-trend** | 176  275  287  228  966 | -0.11 (-0.24; 0.02)  -0.12 (-0.23; -0.01)  -0.08 (-0.19; 0.03)  ref  0.05 | -0.02 (-0.05; 0.02)  -0.04 (-0.07; -0.01)  -0.03 (-0.06; -0.00)  ref  0.21 | 0.08 (-0.10; 0.26)  0.09 (-0.07; 0.25)  0.00 (-0.16; 0.16)  ref  0.21 | -0.02 (-0.05; 0.01)  -0.04 (-0.07; -0.02)  -0.03 (-0.06; -0.00)  ref  0.09 |  |
| **Age at menarche continuous (per 5 years) ^f^** | 966 | 0.16 (0.01, 0.31) | 0.02 (-0.02, 0.06) | -0.12 (-0.34, 0.09) | 0.03 (-0.01, 0.07) |  |
| **Time between menarche and age at first birth, continuous (per 5 years) ^g^** | 866 | 0.02 (-0.04; 0.08) | -0.01 (-0.02; 0.01) | -0.01 (-0.09; 0.06) | -1.4x10^-3^ (-0.02; 0.01) |  |
| **Time since last pregnancy, continuous (per 5 years) ^c^** | 839 | -0.05 (-0.12; 0.02) | -0.02 (-0.04; -2.7x10^-3^) | 0.05 (-0.03; 0.13) | -0.02 (-0.04; -2.7x10^-3^) |  |

^a^ Fibroglandular tissue represents combined epithelium and stroma

**^b^** Adjusted for age (continuous), BMI (continuous), age at menarche (<12, 12, 13, >13), a family history of breast cancer (Yes/No), menopausal status/postmenopausal hormone use (premenopausal, postmenopausal/no hormones, postmenopausal/past hormones, postmenopausal/current hormones, postmenopausal/unknown hormone use status), NHS cohort (NHSI, NHSII), BBD subtype (non-proliferative, proliferative without atypia, and proliferative with atypia), and alcohol use (none, >0-<5, ≥5 g/day)

**^c^** Among parous women only: adjusted for age (continuous), BMI (continuous), age at menarche (<12, 12, 13, >13), parity, age at first child’s birth, a family history of breast cancer (Yes/No), menopausal status/postmenopausal hormone use (premenopausal, postmenopausal/no hormones, postmenopausal/past hormones, postmenopausal/current hormones, postmenopausal/unknown hormone use status), NHS cohort (NHSI, NHSII), BBD subtype (non-proliferative, proliferative without atypia, and proliferative with atypia), and alcohol use (none, >0-<5, ≥5 g/day)

**^d^** Among parous women only: adjusted for age (continuous), BMI (continuous), age at first birth, age at menarche (<12, 12, 13, >13), a family history of breast cancer (Yes/No), menopausal status/postmenopausal hormone use (premenopausal, postmenopausal/no hormones, postmenopausal/past hormones, postmenopausal/current hormones, postmenopausal/unknown hormone use status), NHS cohort (NHSI, NHSII), BBD subtype (non-proliferative, proliferative without atypia, and proliferative with atypia), and alcohol use (none, >0-<5, ≥5 g/day)

**^e^** Among parous women only: adjusted for age (continuous), BMI (continuous), parity, age at menarche (<12, 12, 13, >13), a family history of breast cancer (Yes/No), menopausal status/postmenopausal hormone use (premenopausal, postmenopausal/no hormones, postmenopausal/past hormones, postmenopausal/current hormones, postmenopausal/unknown hormone use status), NHS cohort (NHSI, NHSII), BBD subtype (non-proliferative, proliferative without atypia, and proliferative with atypia), and alcohol use (none, >0-<5, ≥5 g/day)

**^f^** Adjusted for age (continuous), BMI (continuous), parous status (nulliparous, parous), a family history of breast cancer (Yes/No), menopausal status/postmenopausal hormone use (premenopausal, postmenopausal/no hormones, postmenopausal/past hormones, postmenopausal/current hormones, postmenopausal/unknown hormone use status), NHS cohort (NHSI, NHSII), BBD subtype (non-proliferative, proliferative without atypia, and proliferative with atypia), and alcohol use (none, >0-<5, ≥5 g/day)

**^g^** Among parous women only: adjusted for age (continuous), BMI (continuous), parity, a family history of breast cancer (Yes/No), menopausal status/postmenopausal hormone use (premenopausal, postmenopausal/no hormones, postmenopausal/past hormones, postmenopausal/current hormones, postmenopausal/unknown hormone use status), NHS cohort (NHSI, NHSII), BBD subtype (non-proliferative, proliferative without atypia, and proliferative with atypia), and alcohol use (none, >0-<5, ≥5 g/day)

**Supplementary table 5.** **Associations of reproductive variables with percentage of different tissue types (log-transformed) in benign breast biopsy samples of premenopausal women (β coefficients and 95% Confidence Intervals), additionally adjusted for BBD subtype**

| **Reproductive factor** | **N** | **Tissue type** | | | | |
| --- | --- | --- | --- | --- | --- | --- |
|  |  | **% Epithelial** | **% Stroma** | **% Fat** | **% Fibroglandular^a^** |  |
| **Nulliparity^b^**  **Nulliparous**  **Parous** | 61  540 | -0.24 (-0.40; -0.08) ref | 0.06 (0.01; 0.10) ref | -0.30 (-0.54; -0.05) ref | 0.02 (-0.01; 0.06) ref |  |
| **Breastfeeding, months^c^**  **0-<1**  **1-<12**  **12-<24**  **≥24**  **p-trend** | 210  162  92  47  511 | ref  -0.06 (-0.18; 0.06)  -0.03 (-0.18; 0.12)  -0.10 (-0.30; 0.10)  0.42 | ref  0.04 (0.01; 0.07)  0.01 (-0.03; 0.05)  0.04 (-0.02; 0.09)  0.36 | ref  -0.11 (-0.28; 0.07)  -0.01 (-0.22; 0.21)  -0.17 (-0.47; 0.12)  0.45 | ref  0.03 (-0.00; 0.05)  0.00 (-0.04; 0.04)  0.02 (-0.03; 0.07)  0.65 |  |
| **Parity ^d^**  **1**  **2**  **3**  **≥4**  **p-trend** | 62  190  167  108  527 | ref  0.28 (0.12; 0.45)  0.27 (0.10; 0.45)  0.36 (0.18; 0.55)  <0.01 | ref  -0.02 (-0.06; 0.03)  -0.04 (-0.09; 0.01)  -0.05 (-0.11; -0.00)  0.02 | ref  -0.19 (-0.43; 0.05)  -0.06 (-0.31; 0.20)  -0.00 (-0.28; 0.27)  0.35 | ref  0.03 (-0.01; 0.07)  0.00 (-0.04; 0.04)  -0.00 (-0.05; 0.04)  0.32 |  |
| **Parity continuous ^d^** | 527 | 0.07 (0.03; 0.12) | -0.02 (-0.03; -0.00) | 0.02 (-0.04; 0.09) | -0.01 (-0.02; 0.00) |  |
| **Age at first child’s birth ^e^**  **<25**  **25-29**  **≥30**  **p-trend** | 263  205  59  527 | ref  0.20 (0.09; 0.30)  0.27 (0.10; 0.44)  <<0.01 | ref  -0.01 (-0.04; 0.02)  -0.05 (-0.09; 0.00)  0.06 | ref  -0.18 (-0.33; -0.03)  -0.03 (-0.28; 0.22)  0.40 | ref  0.02 (-0.01; 0.04)  -0.00 (-0.04; 0.04)  0.77 |  |
| **Age at first birth continuous (per 5 years) ^e^** | 527 | 0.11 (0.04; 0.18) | -0.01 (-0.03; 0.01) | -0.05 (-0.16; 0.05) | 0.01 (-0.01; 0.02) |  |
| **Age at menarche ^f^**  **<12**  **12**  **13**  **>13**  **p-trend** | 115  174  187  125  601 | -0.11 (-0.27; 0.04)  -0.13 (-0.26; 0.01)  -0.02 (-0.16; 0.11) ref  0.05 | -0.01 (-0.05; 0.03)  -0.02 (-0.06; 0.01)  -0.02 (-0.06; 0.02)  ref  0.60 | 0.13 (-0.10; 0.36)  0.06 (-0.14; 0.26)  -0.00 (-0.20; 0.20) ref  0.21 | -0.02 (-0.05; 0.01)  -0.03 (-0.06; -0.00)  -0.01 (-0.04; 0.02)  ref  0.12 |  |
| **Age at menarche continuous (per 5 years) ^f^** | 601 | 0.17 (-0.01, 0.34) | 0.01 (-0.04, 0.06) | -0.15 (-0.41, 0.11) | 0.03 (-0.01, 0.07) |  |
| **Time between menarche and age at first birth, continuous (per 5 years) ^g^** | 527 | 0.08 (0.01; 0.14) | -0.01 (-0.03; 0.01) | -0.02 (-0.12; 0.08) | 2.2x10^-3^ (-0.01; 0.02) |  |
| **Time since last pregnancy, continuous (per 5 years) ^c^** | 501 | -0.04 (-0.12; 0.04) | -0.02 (-0.04; 3.5x10^-3^) | 0.07 (-0.04; 0.19) | -0.02 (-0.04; 1.8x10^-3^) |  |

^a^ Fibroglandular tissue represents combined epithelium and stroma

**^b^** Adjusted for age (continuous), BMI (continuous), age at menarche (<12, 12, 13, >13), a family history of breast cancer (Yes/No), NHS cohort (NHSI, NHSII), and alcohol use (none, >0-<5, ≥5 g/day)

**^c^** Among parous women only: adjusted for age (continuous), BMI (continuous), age at menarche (<12, 12, 13, >13), parity, age at first child’s birth, a family history of breast cancer (Yes/No), NHS cohort (NHSI, NHSII), BBD subtype (non-proliferative, proliferative without atypia, and proliferative with atypia),and alcohol use (none, >0-<5, ≥5 g/day)

**^d^** Among parous women only: adjusted for age (continuous), BMI (continuous), age at first birth, age at menarche (<12, 12, 13, >13), a family history of breast cancer (Yes/No), NHS cohort (NHSI, NHSII), BBD subtype (non-proliferative, proliferative without atypia, and proliferative with atypia),and alcohol use (none, >0-<5, ≥5 g/day)

**^e^** Among parous women only: adjusted for age (continuous), BMI (continuous), parity, age at menarche (<12, 12, 13, >13), a family history of breast cancer (Yes/No), NHS cohort (NHSI, NHSII), BBD subtype (non-proliferative, proliferative without atypia, and proliferative with atypia),and alcohol use (none, >0-<5, ≥5 g/day)

**^f^** Adjusted for age (continuous), BMI (continuous), parous status (nulliparous, parous), a family history of breast cancer (Yes/No), NHS cohort (NHSI, NHSII), BBD subtype (non-proliferative, proliferative without atypia, and proliferative with atypia),and alcohol use (none, >0-<5, ≥5 g/day)

**^g^** Among parous women only: adjusted for age (continuous), BMI (continuous), parity, a family history of breast cancer (Yes/No), NHS cohort (NHSI, NHSII), BBD subtype (non-proliferative, proliferative without atypia, and proliferative with atypia),and alcohol use (none, >0-<5, ≥5 g/day)
